# Bifunctionalized Allenes. Part XV. Synthesis of 2,5-dihydro-1,2-oxaphospholes by Electrophilic Cyclization Reaction of Phosphorylated α-Hydroxyallenes

**DOI:** 10.3390/molecules190811056

**Published:** 2014-07-29

**Authors:** Ismail E. Ismailov, Ivaylo K. Ivanov, Valerij Ch. Christov

**Affiliations:** Department of Organic Chemistry & Technology, Faculty of Natural Sciences, Konstantin Preslavsky University of Shumen, 115, Universitetska str., BG-9712 Shumen, Bulgaria; E-Mails: ismail78@mail.bg (I.E.I.); iivanov@shu-bg.net (I.K.I.)

**Keywords:** phosphorylated α-hydroxyallenes, electrophilic cyclization, neighbouring group participation, 2,5-dihydro-1,2-oxaphospholes, (1*E*)-2,3-adducts

## Abstract

This paper discusses a reaction of phosphorylated α-hydroxyallenes with protected or unprotected hydroxy groups involving 5-*endo-trig* cyclizations. Various electrophilic reagents such as sulfuryl chloride, bromine, benzenesulfenyl and benzeneselenenyl chlorides have been applied. The paper describes the reaction of 1-hydroxyalkyl-1,2-dienephosphonates with electrophiles that produces 2-methoxy-2-oxo-2,5-dihydro-1,2-oxaphospholes due to the participation of the phosphonate neighbouring group in the cyclization. On the other hand, (1*E*)-alk-1-en-1-yl phosphine oxides were prepared as mixtures with 2,5-dihydro-1,2-oxaphosphol-2-ium halides in a ratio of about 1:2 by chemo-, regio, and stereoselective electrophilic addition to the C^2^-C^3^-double bond in the allene moiety and subsequent concurrent attack of the external (halide anion) and internal (phosphine oxide group) nucleophiles. The paper proposes a possible mechanism that involves cyclization and additional reactions of the phosphorylated α-hydroxyallenes.

## 1. Introduction

Functionalized allenes are considered to be versatile building blocks for organic synthesis and that fact has attracted growing attention during the past four decades [1,2,3,4,5,6,7,8]. The synthetic potential of functionalized allenes has led to the development of new and unique methods applied in the process of constructing various functionalized heterocyclic and carbocyclic systems [9,10,11,12].

The reactivity of allenes is mainly characterized by electrophilic addition reactions where the addition products of the reagent in one and/or other double bond of the allenic system are usually obtained [13,14,15,16,17,18]. Functionalized allenes are also very interesting substrates as a material of choice to study the electrophilic addition reactions on the carbon-carbon double bonds [19,20,21,22,23]. Functional groups linked to the allenic system change considerably the course of the reactions with electrophilic reagents and this is the significant differrence from allenic hydrocarbons. One can see [19,20,21,22,23] that in most cases the reactions proceed with cyclization of the allenic system bearing a functional group leading to heterocyclic compounds. This makes the investigation of functionalized allenes, more specifically the study of their reactions with electrophilic reagents, quite an interesting and topical task.

It is known that the 2,5-dihydrofurans and derivatives thereof represent pivotal structural elements in a wide variety of different biologically active molecules. For instance, they can be found in mycotoxins such as verrucosidine [24] and the structurally related citreoviridine [25] as well as vitamin A metabolites [26], polyether antibiotics [27,28], spiroketals [29], and even amino acids [30]. Thus, the efficient synthesis of suitably functionalized 2,5-dihydrofurans by electrophilic cyclization of α-hydroxyallenes [31,32,33,34,35,36,37,38,39,40,41,42,43,44] is highly attractive.

On the contrary, the literature data on the reactions of phosphorylated allenes (phosphonates, phosphinates and phosphine oxides) with electrophilic reagents reveal that the reactions proceed with cyclization of the allenic system bearing the phosphoryl group (O=P-C=C=C) to give heterocyclic compounds in most cases and the outcome depends on the structure of the starting allenic compound as well as the type of electrophile used [19,20,21,22,23]. The reaction of electrophilic reagents with allenephosphonates [19,20,21,22,23] or allenyl phosphine oxides [45,46,47] leads to 2,5-dihydro-1,2-oxaphospholes or/and 2,1- or/and 2,3-adducts or a mixture of these compounds, depending on the degree of substitution at the C^1^- and C^3^-atoms of the allenic system, as well as on the nature of these substituents, and on the type of the reagents. Ma and coworkers [48,49,50] recently observed that the electrophilic iodohydroxylation [48], fluorohydroxylation [49] and selenohydroxylation [50] reactions of allenyl phosphine oxides with iodine, Selectfluor and benzeneselenenyl chloride lead to 2-iodo-(respectively 2-fluoro- or 2-phenylselenenyl-)3-hydroxy-1(*E*)-alkenyl phosphine oxides with high regio- and stereoselectivities. In [48,49,50] the respective authors comment that this fact is due to the neighbouring group participation effect of the diphenyl phosphine oxide functionality. In recent papers we have reported the reactions of 1-vinyl- [51] and 3-vinylallenyl [52] phosphine oxides with electrophiles leading to formation of various heterocyclic or highly unsaturated compounds.

**Scheme 1:**
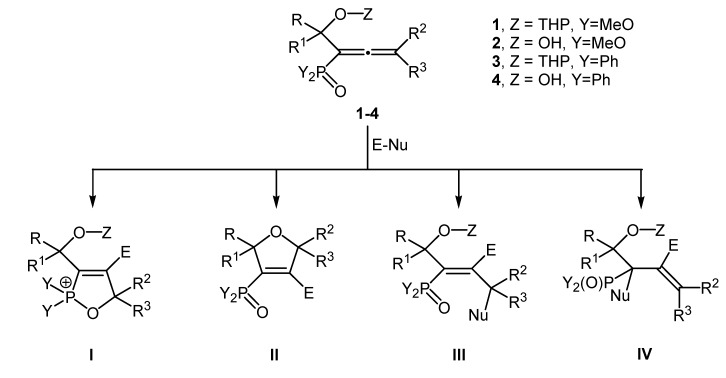
Probable products of the electrophilic reaction of the phosphorylated α-hydroxyallenes **1**–**4**.

Our long-standing research program focuses on the development of efficient electrophilic cyclization reactions of 1,3-bifunctionalized allenes [53,54]. More specifically, our attention is drawn to 1,1-bifunctionalized allenes such as **1**–**4** that comprise a phosphoryl and a hydroxyalkyl group (Scheme 1). The applications of these groups as temporary transformers of chemical reactivity of the allenic system in the synthesis of eventually heterocyclic compounds are of particular interest. These molecules can be considered a combination of an allenephosphonate or allenyl phosphine oxide and a hydroxyallene and they are supposed to have different reactivity profiles in electrophilic reactions. Our recent research has led to a significant result, whereby we have developed a convenient and efficient method for the regioselective synthesis of phosphorylated α-hydroxyallenes using an atom economical [2,3]-sigmatropic rearrangement of intermediate propargyl phosphites or phosphinites, which can be readily prepared via reactions of protected alkynols with dimethyl chlorophosphite or chlorodiphenyl phosphine, respectively, in the presence of a base [55].

## 2. Results and Discussion

### 2.1. Electrophilic Cyclization Reaction of Phosphorylated α-Hydroxyallenes with Protected and Unprotected Hydroxy Groups

It is necessary to draw attention to the fact that conceptually two distinct modes of cyclization of the phosphorylated α-hydroxyallenes are possible. They depend on the electrophilic atom that forms a new bond with the central carbon of the allenic system, which seems likely [19,20,21,22,23]. It is evident that these pathways are closely connected with the intramolecular neighbouring group participation of the phosphoryl and/or the hydroxyalkyl groups as internal nucleophile(s) in the final step of the cyclization. Besides the 5-*endo-trig* cyclizations [56] to the 2,5-dihydro-1,2-oxaphospholes **I** or to the 2,5-dihydrofurans **II**, electrophilic addition might afford the 2,3-adducts **III** and/or the 3,2-adducts **IV ** (Scheme 1).

The present paper is a part of our long-term objective to investigate both the advantages and the limitations of the electrophilic cyclization reactions of 1,1-bifunctionalized allenes.

#### 2.1.1. Electrophilic Cyclization Reaction of the 1-Hydroxyalkyl-allenephosphonates **1** and **2**

**Scheme 2:**
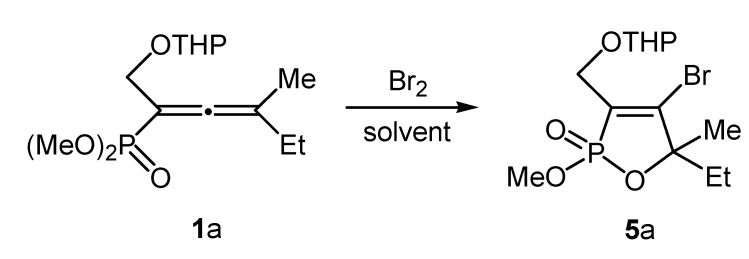
Synthesis of the 2-[(4-bromo-5-ethyl-2-methoxy-5-methyl-2-oxo-2,5-dihydro-1,2-oxaphosphol-3-yl)methoxy]-tetrahydro-2*H*-pyran **5a**.

We started the present study with the electrophilic cyclization reaction of dimethyl 3-methyl-1-(tetrahydro-2*H*-pyran-2-yloxymethyl)-penta-1,2-dienephosphonate (**1a**) with bromine (Scheme 2).

The reaction occurred with cyclization by neighbouring group participation of the phosphonate group with formation of the 2-[(4-bromo-5-ethyl-2-methoxy-5-methyl-2-oxo-2,5-dihydro-1,2-oxaphosphol-3-yl)methoxy]-tetrahydro-2*H*-pyran (**5a**). The cyclization of compound **1a** was already reported by Brel [57], although the range of electrophiles used in that case were limited. We decided to optimize the reaction conditions by studying the electrophile equivalents, reaction temperature, time and solvent effect under an argon atmosphere (Table 1).

**Table 1:** Screening of the reaction conditions for the electrophilic cyclization reaction of the dimethyl 3-methyl-1-(tetrahydro-2*H*-pyran-2-yloxymethyl)-penta-1,2-dienephosphonate **1a** with bromine.

Entry	Bromine (equiv.)	Solvent ^a^	Reaction Temp. (°C)	Reaction Time (h)	Yield ^b^ (%)
1	1.0	CCl_4_	rt	6	45
2	1.0	benzene	rt	8	34
3	1.0	CHCl_3_	rt	5.5	58
4	1.0	CH_2_Cl_2_	rt	5	62
5	1.0	CH_2_Cl_2_	reflux	4	57
**6**	**1.2**	**CH_2_Cl_2_**	**−20**	**3**	**81**
7	1.5	CH_2_Cl_2_	−20	2.5	76
8	2.0	CH_2_Cl_2_	−20	2.5	73
9	1.2	CH_2_Cl_2_	−78	5	77
10	1.2	ClCH_2_CH_2_Cl	−20	4	78
11	1.2	ClCH_2_CH_2_Cl	−30	4	75
12	1.2	MeCN	−20	4.5	68
13	1.2	MeNO_2_	−20	4	72

^a^ Reaction was carried out in the appropriate solvent (10 mL + 10 mL); ^b^ Yields determined by ^1^H and ^31^P NMR analysis.

It should be noted that when the reaction was conducted in nonpolar solvents like CCl_4_ and benzene at room temperature, thin-layer chromatography showed that the two reactants still interacted and the reaction was completed within 6 and 8 h with the formation of the desired product albeit with low yields (45% and 34%, entries 1 and 2). When the reaction was carried out in polar solvent (entries 3–13), it was completed within 2.5–5 h and the yields were considerably higher (57%–81%). Lower yields were obtained at room temperature and reflux (entries 1–5). Fortunately, when sub-zero temperatures were used for 2.5–6 h (entries 6–13), the yield improved to 81% (−20 °C for 3 h, entry 6). Solvents such as 1,2-dichloroethane, acetonitrile, and nitromethane (entries 10–13) gave lower yields, even after longer reaction times (4–4.5 h). When 1.2 equivalents of electrophilic reagent were used, the reaction yields were higher (entries 6, and 9–13). Reactions at −78 and −30 °C for 5 and 4 h gave lower yields (77% and 75%, entries 9 and 11). We therefore, conducted the remainder of the reactions in CH_2_Cl_2_ at −20 °C using 1.0 equivalent of the allenephosphonate with protected hydroxy group **1a** and 1.2 equiv. of the electrophile bromine.

**Scheme 3:**
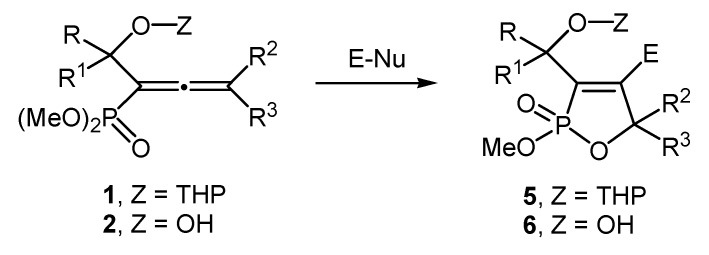
Synthesis of the 2-methoxy-2-oxo-2,5-dihydro-1,2-oxaphospholes **5** and **6**.

When we used the α-hydroxy-allenephosphonate with unprotected hydroxy group **2a** corresponding to **1a** as a starting material, the reaction with bromine under the optimized reaction conditions for 3 h results in the formation of (4-bromo-5-ethyl-2-methoxy-5-methyl-2-oxo-2,5-dihydro-1,2-oxaphosphol-3-yl)-methanol (**6a**) in 80% yield. We used NMR (^1^H-, ^13^C-, and ^31^P-) and IR spectroscopy to reveal the characteristics of the cyclic products **5a** and **6a**. Once we determined the optimized reaction conditions, we focused on the scope of the electrophilic cyclization reaction of the α-hydroxy-allenephosphonates **1a**–**e** and **2a**–**e** with protected and unprotected hydroxy groups (Scheme 3) and the results obtained are summarized in Table 2. We have to say that the reaction under this very set of standard reaction conditions in the favour of 5-*endo-trig* mode affords the 2-methoxy-2-oxo-2,5-dihydro-1,2-oxaphospholes **5a**–**e** and **6a**–**e** to have very good to excellent yields and it does not depend on the nature of the substituents on the allenic system and the hydroxy group, as a result of the neighbouring group participation of phosphonate group in the cyclization. The reaction scope is the following: R and R^1^ can be H or methyl, R^2^ and R^3^ can be methyl, ethyl, butyl, or -(CH_2_)_5_-, E can be Cl, Br, PhS, or PhSe, and Nu-Cl or Br.

**Table 2:** Synthesis of the 2-methoxy-2-oxo-2,5-dihydro-1,2-oxaphospholes **5** and **6**.

	Entry	Allene	R	R^1^	R^2^	R^3^	E	Nu	Product	Time (h)	Yield ^a^ (%)
	1	1a	H	H	Me	Et	Br	Br	5a	3	81
	2	1b	H	H	Me	Bu	Br	Br	5b	3	80
	3	1c	H	H	-(CH_2_)_5_- -(CH_2_)_5_- -(CH_2_)_5_-		Cl	Cl	5c	3	83
	4	1c	H	H			Br	Br	5d	3.5	84
	5	1d	H	Me			PhSe	Cl	5e	4	74
	6	1e	Me	Me	Me	Bu	PhSe	Cl	5f	4.5	73
	7	2a	H	H	Me	Et	Br	Br	6a	3	80
	8	2b	H	H	Me	Bu	PhS	Cl	6b	6	75
	9	2c	H	H	-(CH_2_)_5_-		PhSe	Cl	6c	4.5	74
	10	2d	H	Me	-(CH_2_)_5_-		Cl	Cl	6d	3.5	82
	11	2e	Me	Me	Me	Bu	Br	Br	6e	4	81

^a^ Isolated yields by chromatographic purification.

#### 2.1.2. Concurrent Electrophilic Cyclization and Addition Reactions of 1-Hydroxyalkyl-allenyl Phosphine Oxides **3** and **4**

**Scheme 4:**
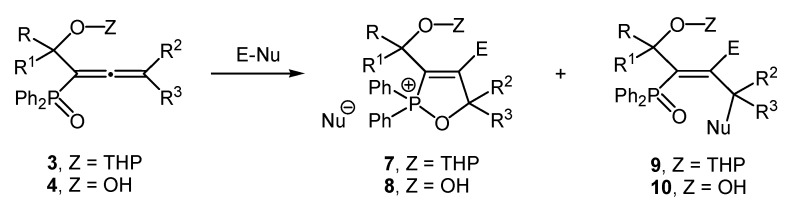
Synthesis of the 2,2-diphenyl-2,5-dihydro-1,2-oxaphosphol-2-ium halides **7** and **8** and the (1*E*)-alk-1-en-1-yl diphenyl phosphine oxides **9** and **10**.

In order to outline the general terms of this methodology, the reaction of the 1-hydroxyalkyl-allenyl phosphine oxides with protected and unprotected hydroxyl group **3** and **4** with different electrophilic reagents such as sulfuryl chloride, bromine, benzenesulfenyl chloride and benzeneselenenyl chloride was thorougly investigated. Surprisingly, once we applied the current standard conditions to the 1,1-bifunctionalized allenes comprising a phosphine oxide and a hydroxyalkyl groups such as **3** and **4** (Scheme 4), the interaction affords mixtures of the 2,2-diphenyl-2,5-dihydro-1,2-oxaphosphol-2-ium halides **7a**–**g** and **9a**–**g** and the (1*E*)-alk-1-en-1-yl diphenyl phosphine oxides **8a**–**e** and **10a**–**e** in the ratio about 2:1 in 70%–80% total yield after stirring for several h at −20 °C and for one hour to rt.

**Table 3:** Synthesis of the 2,2-diphenyl-2,5-dihydro-1,2-oxaphosphol-2-ium halides **7** and **8** and the (1*E*)-alk-1-en-1-yl diphenyl phosphine oxides **9** and **10**.

Entry	Allene	R	R^1^	R^2^	R^3^	E	Nu	Time, h	Products (Yield ^a^ %)		Ratio
1	3a	H	H	Me	Et	Br	Br	3	7a (50)	9a (23)	2.17:1
2	3b	H	H	Me	Bu	Br	Br	4	7b (48)	9b (22)	2.18:1
3	3c	H	H	-(CH_2_)_5_- -(CH_2_)_5_- -(CH_2_)_5_-		PhSe	Cl	5.5	7c (50)	9c (24)	2.08:1
4	3c	H	H			Br	Br	3.5	7d (49)	9d (24)	2.04:1
5	3d	H	Me			Br	Br	4	7e (49)	9e (25)	1.96:1
6	3d	H	Me	-(CH_2_)_5_-		PhSe	Cl	5	7f (48)	9f (24)	2.00:1
7	3e	Me	Me	Me	Bu	Cl	Cl	4	7g (54)	9g (25)	2.16:1
8	4a	H	H	Me	Et	Br	Br	2.5	8a (52)	10a (23)	2.20:1
9	4b	H	H	Me	Bu	PhSe	Cl	5	8b (46)	10b (26)	1.78:1
10	4c	H	H	-(CH_2_)_5_- -(CH_2_)_5_-		Cl	Cl	3	8c (54)	10c (26)	2.11:1
11	4d	H	Me			PhS	Cl	8	8d (45)	10d (25)	1.84:1
12	4e	Me	Me	Me	Bu	PhSe	Cl	6.5	8e (46)	10e (24)	1.90:1

^a^ Isolated yields by chromatographic purification.

The results are summarized in Table 3. These reaction pathways may be interpreted as a result of the concurrent neighbouring group participation of the phosphonate group as an internal nucleophile to give cyclic products **7a**–**g** and **9a**–**g** and the highly regio- and stereoselective association of the external nucleophile, indicating a highly chemoselectively addition reaction of the electrophilic reagents to the C^2^-C^3^-double bond of the allenic system with formation of the 1*E*-2,3-adducts **8a**–**e** and **10a**–**e**.

Thus, the reaction of phosphorylated α-hydroxyallenes with protected or unprotected hydroxy groups with different electrophilic reagents occurs via 5-*endo-trig* cyclization. Treatment of the 1-hydroxyalkyl-allenephosphonates **1** and **2** with electrophiles gives the 2-methoxy-2-oxo-2,5-dihydro-1,2-oxaphospholes **5** and **6** as a result of the neighbouring group participation of the phosphonate group in the cyclization, while the (1*E*)-alk-1-en-1-yl phosphine oxides **9** and **10** were prepared as mixtures with the 2,5-dihydro-1,2-oxaphosphol-2-ium halides **7** and **8** in a ratio of about 1:2 by chemo, regio, and stereoselective electrophilic addition to the C^2^-C^3^-double bond in the allene moiety and subsequent concurrent attack of the external (halide anion) and internal (phosphine oxide group) nucleophiles.

### 2.2. A Rationale for the Reaction of the Phosphorylated α-Hydroxyallenes **1**–**4** with Electrophilic Reagents

**Scheme 5:**
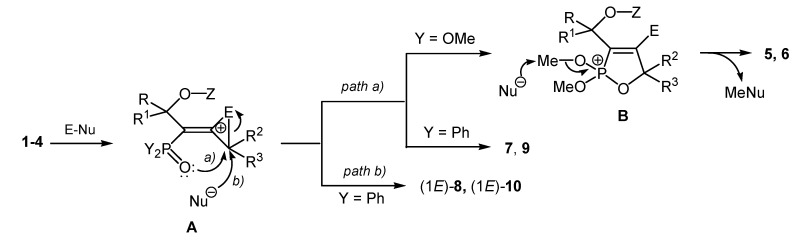
A rationale for the reaction of the phosphorylated α-hydroxyallenes **1**–**4** with electrophilic reagents.

A rationale for this reaction based on available literature data [13,14,15,16,17,18,19,20,21,22,23] and on our recent results [51,52,53,54] is depicted in Scheme 5.

The starting point is the attack of the electrophile (Cl^+^, Br^+^, S^+^ or Se^+^) on the most nucleophilic atom of the allenic system of π-bonds (C^3^) with formation of the cyclic onium (chloronium, bromonium, thiiranium or seleniranium) ions **A** after attack on the relatively more electron-rich C^2^-C^3^-double bond. Then the ions **A** are easily transformed into the more stable five-membered cyclic ions **B** via the attachment of the oxygen atom of the phosphonate functionality (*path a*). Further, the intermediates **B** undergo nucleophilic attack on the MeO group and elimination of methyl halide (MeNu) affording the final cyclic products **5** and **6** (when Y is OMe). On the other hand, in the case where the 1-hydroxyalkyl-allenyl phosphine oxides **3** and **4** are used as starting materials (Y is Ph), the formation of the final 2,2-diphenyl-2,5-dihydro-1,2-oxaphosphol-2-ium halides **7** and **9** takes placesince the elimination of an methyl halide (second stage of an Arbuzov type rearrangement) and formation of products with tetracoordinated phosphorus is impossible. The preparation of the(1*E*)-alk-1-en-1-yl phosphine oxides **8** and **10 **as mixtures with the cyclic phosphonium halides **7** and **9 **in a ratio of about 1:2 can be considered in terms of the assumption of a concurrent attack of the external nucleophile on the cyclic three-membered onium ion **A** (*path b*). The stereoselectivity could be explained by the favorable *trans* arrangement of the electrophile and the phosphine oxide group and *anti*-attack of the external nucleophile Nu on the onium ion **A**. This is supposed to arise from attack on the allenic C^2^-C^3^ double bond *anti* to the phosphoryl group which assists in the cyclization by neighbouring group participation as an internal nucleophile.

The abovementioned explanation should account for the results on the study of the reactions of other bifunctionalized allenes with electrophilic reagents and, more specifically, their stereochemistry. Further work in this area shall focus on exploiting and extending the synthetic utility of the 1,1-bifunctionalized allenes for the preparation of different heterocyclic systems by application of the electrophilic cyclization methodology.

## 3. Experimental Section

### 3.1. General Information

All new synthesized compounds were purified by column chromatography and characterized on the basis of NMR, IR, and microanalytical data. NMR spectra were recorded on DRX Bruker Avance-250 (^1^H at 250.1 MHz, ^13^C at 62.9 MHz, ^31^P at 101.2 MHz) and Bruker Avance II + 600 (Bruker BioSpinGmbH, Karlsruhe, Germany) (^1^H at 600.1 MHz, ^13^C at 150.9 MHz, ^31^P at 242.9 MHz) spectrometers for solutions in CDCl_3_. All ^1^H-and ^13^C-NMR experiments were measured referring to the signal of internal TMS and ^31^P-NMR experiments were measured referring to the signal of external 85% H_3_PO_4_. *J* values are given in hertz. IR spectra were recorded with an FT-IR_Afinity-1 Shimadzu spectrophotometer (Shimadzu, Tokyo, Japan). Elemental analyses were carried out by the Microanalytical Service Laboratory of Faculty of Chemistry and Pharmacy, University of Sofia, Bulgaria, using Vario EL*3* CHNS(O) (Elementar Analysensysteme, Hanau, Germany). Column chromatography was performed on Kieselgel F_254_ 60 (70–230 mesh ASTM, 0.063–0.200 nm, Merck, Darmstadt, Germany). CH_2_Cl_2_ was distilled over CaH_2_ and other commercially available chemicals were used without additional purification unless otherwise noted. Reactions were carried out in oven dried glassware under an argon atmosphere and exclusion of moisture. All compounds were checked for purity on Kieselgel F_254_ 60 TLC plates (Merck).

### 3.2. Starting Materials

Diphenyl disulfide and sulfuryl chloride in dichloromethane and distilled *in vacuo* (bp 80–81 °C/20 mm Hg) [58] were used to prepare benzenesulfanyl chloride. Diphenyl disulfide, sulfuryl chloride, and benzeneselenenyl chloride were commercially available and used without purification. The starting phosphorylated α-hydroxyallenes **1**–**4** were prepared according to the established procedure [55].

### 3.3. General Procedure for the Reactions of the Dimethyl 1-(Tetrahydro-2H-pyran-2-yloxy)methyl-1,2-dienephosphonates **1** with Electrophilic Reagents

To a solution of the dimethyl 1-(tetrahydro-2*H*-pyran-2-yloxy)methyl-1,2-dienephosphonates **1** (3.0 mmol) in dry dichloromethane (10 mL) at −20 °C was added dropwise with stirring a solution of electrophilic reagent (sulfuryl chloride, bromine or benzeneselenenyl chloride) (3.6 mmol) in the same solvent (10 mL). The reaction mixture was stirred at the same temperature for several h (see Table 1) and an hour at room temperature. After evaporation of the solvent, the residue was chromatographed on a silica gel column (ethyl acetate and hexane 4:1) as eluent to give the pure products **5** as oils, which had the following properties:

*2-[(4-Bromo-5-ethyl-2-methoxy-5-methyl-2-oxo-2,5-dihydro-1,2-oxaphosphol-3-yl)methoxy]-tetra-hydro-2H-pyran* (**5a**). Yellow oil, yield: 81%. R_f_ 0.49; IR (neat, cm^−1^): 1015 (C-O-P), 1120 (C-O-C), 1268 (P=O), 1583 (C=C). ^1^H-NMR (250.1 MHz): δ 0.89 (t, *J =* 7.2 Hz, 3H, Me-CH_2_), 1.53 (s, 3H, Me-C), 1.54–1.89, 3.55–3.68, 4.53–4.63 (overlapping multiplets, 9H, OTHP), 1.77–1.88 (m, 2H, Me-CH_2_), 3.83 (d, *J =* 9.3 Hz, 3H, MeO), 3.91–4.07 (m, 2H, CH_2_O). ^13^C-NMR (62.9 MHz) δ = 9.3 (*J =* 4.7 Hz), 19.6, 24.6 (*J =* 7.7 Hz), 31.2, 31.5 (*J =* 7.9 Hz), 32.5 (*J =* 7.8 Hz), 53.4 (*J =* 13.9 Hz), 63.7, 65.8 (*J =* 5.7 Hz), 89.6 (*J =* 9.8 Hz), 97.1 (*J =* 5.0 Hz), 130.5 (*J =* 156.4 Hz), 140.7 (*J =* 51.4 Hz). ^31^P-NMR (101.2 MHz): δ 31.8. Anal. Calcd for C_13_H_22_BrO_5_P (369.19): C 42.29, H 6.01. Found: C 42.35, H 5.93.

*2-[(4-Bromo-5-butyl-2-methoxy-5-methyl-2-oxo-2,5-dihydro-1,2-oxaphosphol-3-yl)methoxy]-tetra-hydro-2H-pyran* (**5b**). Dark orange oil, yield: 80%. R_f_ 0.53; IR (neat, cm^−1^): 1012 (C-O-P), 1123 (C-O-C), 1263 (P=O), 1587 (C=C). ^1^H-NMR (600.1 MHz): δ 0.91 (t, *J =* 7.3 Hz, 3H, Me-CH_2_), 1.28–1.36, 1.49–1.60, 1.77–1.85 (overlapping multiplets, 6H, (CH_2_)_3_-Me), 1.51–1.58, 3.56–3.60, 4.54–4.63 (overlapping multiplets, 9H, OTHP), 1.56 (s, 3H, Me-C), 3.85 (d, *J =* 9.4 Hz, 3H, MeO), 3.89–3.99 (m, 2H, CH_2_O). ^13^C-NMR (150.9 MHz) δ = 14.1, 19.9, 22.6, 23.4 (*J =* 4.6 Hz), 25.1 (*J =* 7.9 Hz), 26.7, 32.5, 40.5 (*J =* 7.9 Hz), 53.2 (*J =* 14.2 Hz), 62.8, 64.9 (*J =* 5.6 Hz), 88.7 (*J =* 10.0 Hz), 96.5 (*J =* 5.1 Hz), 129.9 (*J =* 155.6 Hz), 141.5 (*J =* 52.1 Hz). ^31^P-NMR (242.9 MHz): δ 31.9. Anal. Calcd for C_15_H_26_ BrO_5_P (397.24): C 45.35, H 6.60. Found: C 45.29, H 6.56.

*4-Chloro-2-methoxy-3-[(tetrahydro-2H-pyran-2-yloxy)methyl]-1-oxa-2-phosphaspiro[4.5]dec-3-ene 2-oxide* (**5c**). Yellow oil, yield: 83%. R_f_ 0.47; IR (neat, cm^−1^): 1019 (C-O-P), 1117 (C-O-C), 1261 (P=O), 1584 (C=C). ^1^H-NMR (250.1 MHz): δ 1.32–1.92, 2.14–2.23, 3.61–3.77, 4.53–4.59 (overlapping multiplets, 19H, (CH_2_)_5_, OTHP), 3.69 (d, *J =* 9.4 Hz, 3H, MeO), 3.95–4.07 (m, 2H, CH_2_O). ^13^C-NMR (62.9 MHz) δ = 19.5, 22.6 (*J =* 5.0 Hz), 24.1, 25.7, 31.7, 35.4 (*J =* 7.8 Hz), 36.5 (*J =* 7.7 Hz), 52.4 (*J =* 14.5 Hz), 62.4, 64.6 (*J =* 5.7 Hz), 87.2 (*J =* 9.5 Hz), 96.5 (*J =* 4.9 Hz), 129.5 (*J =* 156.4 Hz), 140.7 (*J =* 52.5 Hz). ^31^P-NMR (101.2 MHz): δ 32.4. Anal. Calcd for C_15_H_24_ClO_5_P (350.77): C 51.36, H 6.90. Found: C 51.43, H 6.96.

*4-Bromo-2-methoxy-3-[(tetrahydro-2H-pyran-2-yloxy)methyl]-1-oxa-2-phosphaspiro[4.5]dec-3-ene 2-oxide* (**5d**). Dark orange oil, yield: 84%. R_f_ 0.51; IR (neat, cm^−1^): 1013 (C-O-P), 1117 (C-O-C), 1269 (P=O), 1581 (C=C). ^1^H-NMR (600.1 MHz): δ 1.29–1.68, 1.95–2.05, 2.28–2.36, 3.60–3.76, 4.52–4.57 (overlapping multiplets, 19H, (CH_2_)_5_, OTHP), 3.78 (d, *J =* 9.3 Hz, 3H, MeO), 3.93–4.06 (m, 2H, CH_2_O). ^13^C-NMR (150.9 MHz) δ = 19.3, 22.0 (*J =* 4.8 Hz), 23.9, 25.4, 31.5, 34.6 (*J =* 7.9 Hz), 37.1 (*J =* 7.9 Hz), 52.5 (*J =* 14.4 Hz), 62.2, 64.8 (*J =* 5.9 Hz), 87.1 (*J =* 9.7 Hz), 96.3 (*J =* 5.0 Hz), 129.2 (*J =* 156.0 Hz), 139.6 (*J =* 51.6 Hz). ^31^P-NMR (242.9 MHz): δ 33.0. Anal. Calcd for C_15_H_24_BrO_5_P (395.23): C 45.58, H 6.12. Found: C 45.63, H 6.19.

*2-Methoxy-4-phenylselenenyl-3-[1-(tetrahydro-2H-pyran-2-yloxy)ethyl]-1-oxa-2-phosphaspiro[4.5]dec-3-ene 2-oxide* (**5e**). Orange oil, yield: 74%. R_f_ 0.48; IR (neat, cm^−1^): 1011 (C-O-P), 1122 (C-O-C), 1259 (P=O), 1589 (C=C). ^1^H-NMR (600.1 MHz): δ 1.13–1.74, 2.01–2.09, 3.59–3.69, 4.63–4.68 (overlapping multiplets, 19H, (CH_2_)_5_, OTHP), 1.38 (dd, *J =* 10.6 Hz, *J =* 6.5 Hz, 3H, Me-CH), 3.72 (d, *J =* 9.2 Hz, 3H, MeO), 4.21–4.29 (m, 1H, Me-CH), 7.39–7.44 (m, 5H, Ph). ^13^C-NMR (150.9 MHz) δ = 19.4, 21.0 (*J =* 5.0 Hz), 21.3 (*J =* 7.8 Hz), 23.7, 25.6, 31.4, 34.1 (*J =* 7.8 Hz), 36.3 (*J =* 7.9 Hz), 51.9 (*J =* 14.7 Hz), 62.5, 76.2 (*J =* 6.1 Hz), 89.4 (*J =* 9.8 Hz), 95.2 (*J =* 4.9 Hz), 129.4-139.0, 131.4 (*J =* 105.4 Hz), 174.2 (*J =* 81.4 Hz). ^31^P-NMR (242.9 MHz): δ 34.5. Anal. Calcd for C_22_H_31_O_5_PSe (485.41): C 54.44, H 6.44. Found: C 54.40, H 6.52.

*2[1-(5-Butyl-2-methoxy-5-methyl-2-oxo-4-phenylselenenyl-2,5-dihydro-1,2-oxaphosphol-3-yl)methyl-ethoxy]-tetrahydro-2H-pyran* (**5f**). Yellow oil, yield: 73%. R_f_ 0.47; IR (neat, cm^−1^): 1014 (C-O-P), 1121 (C-O-C), 1266 (P=O), 1586 (C=C). ^1^H-NMR (600.1 MHz): δ 0.81 (t, *J =* 7.4 Hz, 3H, Me-CH_2_), 1.26–1.33, 1.39–1.46, 1.81–1.93 (overlapping multiplets, 6H, (CH_2_)_3_-Me), 1.52–1.70, 3.72–3.86, 4.71–4.76 (overlapping multiplets, 9H, OTHP), 1.56 (s, 3H, Me-C), 3.84 (d, *J =* 9.6 Hz, 3H, MeO), 1.48, 1.53 (ss, 6H, Me_2_C), 7.49–7.58 (m, 5H, Ph). ^13^C-NMR (150.9 MHz) δ = 14.2, 20.4, 23.1, 23.6 (*J =* 4.7 Hz), 24.7 (*J =* 8.0 Hz), 25.2, 29.9 (*J =* 7.9 Hz), 32.4, 39.7 (*J =* 8.1 Hz), 52.4 (*J =* 15.0 Hz), 63.7, 84.2 (*J =* 6.0 Hz), 91.7 (*J =* 10.0 Hz), 94.4 (*J =* 4.8 Hz), 128.7–138.7, 132.7 (*J =* 106.9 Hz), 175.4 (*J =* 82.8 Hz). ^31^P-NMR (242.9 MHz): δ 33.5. Anal. Calcd for C_23_H_35_O_5_PSe (501.45): C 55.09, H 7.04. Found: C 55.02, H 6.99.

### 3.4. General Procedure for the Reactions of the 1-Hydroxyalkyl-1,2-dienephosphonates **2** with Electrophilic Reagents

We got a solution of the 1-hydroxyalkyl-1,2-dienephosphonates **2** (3.0 mmol) where in dry dichloromethane (10 mL) at −20 °C was added dropwise with stirring a solution of electrophilic reagent (sulfuryl chloride, bromine, benzenesulfenyl chloride or benzeneselenenyl chloride) (3.6 mmol) in the same solvent (10 mL). The mixture was stirred at the same temperature for several h (see Table 1) and an hour at room temperature. After evaporation of the solvent, the residue was chromatographed on a silica gel column (ethyl acetate and hexane 2:1) as eluent to give the pure products **6 **as oils, which had the following properties:

*(4-Bromo-5-ethyl-2-methoxy-5-methyl-2-oxo-2,5-dihydro-1,2-oxaphosphol-3-yl)-methanol* (**6a**). Yellow oil, yield: 80%. R_f_ 0.56; IR (neat, cm^−1^): 1018 (C-O-P), 1263 (P=O), 1587 (C=C), 3413 (OH). ^1^H-NMR (600.1 MHz): δ 0.89 (t, *J =* 7.1 Hz, 3H, Me-CH_2_), 1.59 (s, 3H, Me-C), 1.78–1.99 (m, 2H, Me-CH_2_), 3.11 (s, 1H, OH), 3.79 (d, *J =* 9.6 Hz, 3H, MeO), 4.51–4.56 (m, 2H, CH_2_O). ^13^C-NMR (150.9 MHz) δ = 9.4 (*J =* 4.8 Hz), 24.3 (*J =* 7.8 Hz), 31.1 (*J =* 7.8 Hz), 52.7 (*J =* 14.3 Hz), 61.4 (*J =* 5.9 Hz), 91.4 (*J =* 9.9 Hz), 129.7 (*J =* 155.0 Hz), 140.4 (*J =* 50.7 Hz). ^31^P-NMR (242.9 MHz): δ 35.7. Anal. Calcd for C_8_H_14_BrO_4_P (285.07): C 33.71, H 4.95. Found: C 33.65, H 5.02.

*(5-Butyl-2-methoxy-5-methyl-2-oxo-4-phenylsulfenyl-2,5-dihydro-1,2-oxaphosphol-3-yl)-methanol* (**6b**). Orange oil, yield: 75%. R_f_ 0.49; IR (neat, cm^−1^): 1010 (C-O-P), 1260 (P=O), 1584 (C=C), 3409 (OH). ^1^H-NMR (600.1 MHz): δ 0.91 (t, *J =* 7.2 Hz, 3H, Me-CH_2_), 1.26–1.35, 1.60–1.64, 1.86–2.05 (overlapping multiplets, 6H, (CH_2_)_3_-Me), 1.47 (s, 3H, Me-C), 3.69 (s, 1H, OH), 3.75 (d, *J =* 9.5 Hz, 3H, MeO), 4.68–4.71 (m, 2H, CH_2_O), 7.16–7.44 (m, 5H, Ph). ^13^C-NMR (150.9 MHz) δ = 14.1, 23.2, 24.4 (*J =* 4.7 Hz), 27.8 (*J =* 7.9 Hz), 40.4 (*J =* 7.8 Hz), 51.9 (*J =* 14.6 Hz), 61.6 (*J =* 6.0 Hz), 88.9 (*J =* 9.8 Hz), 126.7–135.8, 128.6 (*J =* 102.0 Hz), 158.1 (*J =* 51.2 Hz). ^31^P-NMR (242.9 MHz): δ 33.1. Anal. Calcd for C_16_H_23_O_4_PS (342.39): C 56.13, H 6.77. Found: C 56.19, H 6.84.

*(2-Methoxy-2-oxo-4-phenylselenenyl-1-oxa-phospha-spiro[4.5]dec-3-en-3-yl)-methanol* (**6c**). Orange oil, yield: 74%. R_f_ 0.48; IR (neat, cm^−1^): 1018 (C-O-P), 1268 (P=O), 1580 (C=C), 3418 (OH). ^1^H-NMR (250.1 MHz): δ 1.16–1.39, 1.60–1.79, 1.84–2.07 (overlapping multiplets, 10H, (CH_2_)_5_), 3.75 (s, 1H, OH), 3.78 (d, *J =* 9.8 Hz, 3H, MeO), 4.66–4.69 (m, 2H, CH_2_O), 7.28–7.37 (m, 5H, Ph). ^13^C-NMR (62.9 MHz) δ = 21.4 (*J =* 4.9 Hz), 23.9, 33.9 (*J =* 7.8 Hz), 36.2 (*J =* 7.9 Hz), 52.4 (*J =* 14.7 Hz), 60.9 (*J =* 6.0 Hz), 89.4 (*J =* 9.8 Hz), 127.4 (*J =* 106.0 Hz), 127.6–137.9, 174.2 (*J =* 82.4 Hz). ^31^P-NMR (101.2 MHz): δ 36.3. Anal. Calcd for C_16_H_21_O_4_PSe (387.27): C 49.62, H 5.47. Found: C 49.56, H 5.51.

*1-(4-Chloro-2-methoxy-2-oxo-1-oxa-phospha-spiro[4.5]dec-3-en-3-yl)-ethanol* (**6d**). Yellow oil, yield: 82%. R_f_ 0.54; IR (neat, cm^−1^): 1011 (C-O-P), 1259 (P=O), 1583 (C=C), 3424 (OH). ^1^H-NMR (250.1 MHz): δ 1.33–1.48, 1.64–1.85, 1.94–2.14 (overlapping multiplets, 10H, (CH_2_)_5_), 1.48 (dd, *J =* 10.5 Hz, *J =* 6.4 Hz, 3H, Me-CH), 3.67 (d, *J =* 9.4 Hz, 3H, MeO), 3.90 (s, 1H, OH), 4.69–4.78 (m, 1H, Me-CH). ^13^C-NMR (62.9 MHz) δ = 22.4 (*J =* 5.0 Hz), 24.1, 24.5 (*J =* 7.9 Hz), 34.4 (*J =* 7.9 Hz), 36.8 (*J =* 7.9 Hz), 51.9 (*J =* 15.1 Hz), 72.6 (*J =* 5.8 Hz), 90.5 (*J =* 10.1 Hz), 129.3 (*J =* 101.6 Hz), 160.6 (*J =* 40.7 Hz). ^31^P-NMR (101.2 MHz): δ 35.7. Anal. Calcd for C_11_H_18_ClO_4_P (280.68): C 47.07, H 6.46. Found: C 46.99, H 6.40.

*2-(4-Bromo-5-butyl-2-methoxy-5-methyl-2-oxo-2,5-dihydro-1,2-oxaphosphol-3-yl)-propan-2-ol* (**6e**). Dark orange oil, yield: 81%. R_f_ 0.51; IR (neat, cm^−1^): 1009 (C-O-P), 1268 (P=O), 1589 (C=C), 3410 (OH). ^1^H-NMR (600.1 MHz): δ 0.91 (t, *J =* 7.3 Hz, 3H, Me-CH_2_), 1.27–1.34, 1.50–1.57, 1.76–1.92 (overlapping multiplets, 6H, (CH_2_)_3_-Me), 1.48 (s, 3H, Me-C), 1.56, 1.58 (ss, 6H, Me_2_C), 3.48 (s, 1H, OH), 3.65 (d, *J =* 9.5 Hz, 3H, MeO). ^13^C-NMR (150.9 MHz) δ = 14.2, 23.1, 23.5 (*J =* 4.6 Hz), 25.4 (*J =* 7.9 Hz), 31.4 (*J =* 8.1 Hz), 39.4 (*J =* 7.9 Hz), 52.3 (*J =* 15.0 Hz), 71.4 (*J =* 6.0 Hz), 92.4 (*J =* 9.8 Hz), 133.1 (*J =* 154.3 Hz), 142.8 (*J =* 51.4 Hz). ^31^P-NMR (242.9 MHz): δ 34.1. Anal. Calcd for C_12_H_22_BrO_4_P (341.18): C 42.24, H 6.50. Found: C 42.31, H 6.56.

### 3.5. General Procedure for the Reactions of the 2-[2-(Diphenylphosphinoyl)-2,3-dienyloxy]methyl-tetrahydro-2H-pyrans **3** with Electrophilic Reagents

To a solution of the 2-[2-(diphenylphosphinoyl)-2,3-dienyloxy]methyl-tetrahydro-2*H*-pyrans **3** (3.0 mmol) in dry dichloromethane (10 mL) at −20 °C was added dropwise with stirring a solution of electrophilic reagent (sulfuryl chloride, bromine or benzeneselenenyl chloride) (3.6 mmol) in the same solvent (10 mL). The reaction mixture was stirred at the same temperature for several h (see Table 2) and an hour at room temperature. The solvent was removed using a rotatory evaporator and the residue was purified by column chromatography (silica gel, ethyl acetate and hexane 4:1). The pure products **7** and **9** had the following properties:

*4-Bromo-5-ethyl-5-methyl-2,2-diphenyl-3-[(tetrahydro-2H-pyran-2-yloxy)methyl]-2,5-dihydro-1,2-oxaphosphol-2-ium bromide* (**7a**). Orange oil, yield: 50%. R_f_ 0.38; IR (neat, cm^−1^): 1119 (C-O-C), 1439, 1484 (Ph), 1583 (C=C). ^1^H-NMR (600.1 MHz): δ 1.07 (t, *J =* 7.1 Hz, 3H, Me-CH_2_), 1.44–1.69, 3.61–3.75, 4.80–4.85 (overlapping multiplets, 9H, OTHP), 1.78 (s, 3H, Me-C), 2.28–2.37 (m, 2H, Me-CH_2_), 4.31–4.51 (m, 2H, CH_2_O), 7.75–8.46 (m, 10H, 2Ph). ^13^C-NMR (150.9 MHz) δ = 7.9 (*J =* 4.5 Hz), 19.4, 25.4, 27.1 (*J =* 7.9 Hz), 31.2, 31.6 (*J =* 8.0 Hz), 62.6 (*J =* 7.8 Hz), 63.0, 92.4 (*J =* 10.1 Hz), 98.4 (*J =* 4.6 Hz), 111.4–135.2, 134.7 (*J =* 51.2 Hz), 158.7 (*J =* 50.9 Hz). ^31^P-NMR (242.9 MHz): δ 86.5. Anal. Calcd for C_24_H_29_Br_2_O_3_P (556.27): C 51.82, H 5.25. Found: C 51.74, H 5.20.

*(1E)-2,3-Dibromo-3-methyl-1-[(tetrahydro-2H-pyran-2-yloxy)methyl]pent-1-en-1-yl diphenyl phosphine oxide* (**9a**). Colourless oil, yield: 23%. R_f_ 0.62; IR (neat, cm^−1^): 1121 (C-O-C), 1153 (P=O), 1435, 1488 (Ph), 1618 (C=C). ^1^H-NMR (600.1 MHz): δ 1.12 (t, *J =* 7.3 Hz, 3H, Me-CH_2_), 1.46–1.71, 3.62–3.77, 4.51–4.57 (overlapping multiplets, 9H, OTHP), 1.98–2.20 (m, 2H, Me-CH_2_), 2.16 (s, 3H, Me-C), 3.91–4.07 (m, 2H, CH_2_O), 7.53–8.12 (m, 10H, 2Ph). ^13^C-NMR (150.9 MHz) δ = 9.4, 19.6, 25.3, 30.8, 35.4 (*J =* 4.6 Hz), 36.4 (*J =* 5.0 Hz), 62.4, 62.6 (*J =* 7.9 Hz), 68.4 (*J =* 5.8 Hz), 96.3 (*J =* 5.0 Hz), 129.3–133.3, 132.4 (*J =* 154.7 Hz), 141.7 (*J =* 50.4 Hz). ^31^P-NMR (242.9 MHz): δ 39.7. Anal. Calcd for C_24_H_29_Br_2_O_3_P (556.27): C 51.82, H 5.25. Found: C 51.87, H 5.17.

*4-Bromo-5-butyl-5-methyl-2,2-diphenyl-3-[(tetrahydro-2H-pyran-2-yloxy)methyl]-2,5-dihydro-1,2-oxaphosphol-2-ium bromide* (**7b**). Orange oil, yield: 48%. R_f_ 0.37; IR (neat, cm^−1^): 1120 (C-O-C), 1434, 1489 (Ph), 1588 (C=C). ^1^H-NMR (600.1 MHz): δ 0.91 (t, *J =* 6.3 Hz, 3H, Me-CH_2_), 1.08–1.15, 1.28–1.38, 2.26–2.44 (overlapping multiplets, 6H, (CH_2_)_3_-Me), 1.46–1.70, 3.58–3.72, 4.77–4.81 (overlapping multiplets, 9H, OTHP), 1.77 (s, 3H, Me-C), 4.35–4.49 (m, 2H, CH_2_O), 7.73–8.50 (m, 10H, 2Ph). ^13^C-NMR (150.9 MHz) δ = 14.4, 19.5, 23.0, 23.4 (*J =* 4.5 Hz), 25.2, 27.3 (*J =* 8.0 Hz), 31.4, 39.7 (*J =* 7.7 Hz), 62.3, 62.8 (*J =* 7.5 Hz), 92.2 (*J =* 9.8 Hz), 98.3 (*J =* 4.7 Hz), 110.2-134.8, 133.6 (*J =* 49.5 Hz), 159.8 (*J =* 51.2 Hz). ^31^P-NMR (242.9 MHz): δ 86.6. Anal. Calcd for C_26_H_33_Br_2_O_3_P (584.32): C 53.44, H 5.69. Found: C 53.37, H 5.73.

*(1E)-2,3-Dibromo-3-methyl-1-[(tetrahydro-2H-pyran-2-yloxy)methyl]hept-1-en-1-yl diphenyl phosphine oxide* (**9b**). Yellow oil, yield: 22%. R_f_ 0.64; IR (neat, cm^−1^): 1119 (C-O-C), 1163 (P=O), 1439, 1484 (Ph), 1612 (C=C). ^1^H-NMR (600.1 MHz): δ 0.85 (t, *J =* 6.3 Hz, 3H, Me-CH_2_), 1.30–1.43, 1.47–1.72 (overlapping multiplets, 6H, (CH_2_)_3_-Me), 2.01–2.21, 3.36–3.74, 4.52–4.58 (overlapping multiplets, 9H, OTHP), 2.14 (s, 3H, Me-C), 3.92–4.06 (m, 2H, CH_2_O), 7.51–8.13 (m, 10H, 2Ph). ^13^C-NMR (150.9 MHz) δ = 14.4, 19.4, 22.3, 25.4, 27.3, 31.2, 35.2 (*J =* 4.7 Hz), 43.2 (*J =* 5.1 Hz), 58.9 (*J =* 7.8 Hz), 62.4, 67.8 (*J =* 5.9 Hz), 96.4 (*J =* 5.1 Hz), 129.7–134.0, 132.5 (*J =* 155.3 Hz), 142.1 (*J =* 50.9 Hz). ^31^P-NMR (242.9 MHz): δ 39.7. Anal. Calcd for C_26_H_33_Br_2_O_3_P (584.32): C 53.44, H 5.69. Found: C 53.50, H 5.76.

*5-Butyl-5-methyl-2,2-diphenyl-4-phenylselenenyl-3-[(tetrahydro-2H-pyran-2-yloxy)methyl]-2,5-dihydro-1,2-oxaphosphol-2-ium chloride* (**7c**). Orange oil, yield: 50%. R_f_ 0.35; IR (neat, cm^−1^): 1120 (C-O-C), 1444, 1487 (Ph), 1585 (C=C). ^1^H-NMR (600.1 MHz): δ 0.88 (t, *J =* 6.4 Hz, 3H, Me-CH_2_), 1.02–1.11, 1.24–1.36, 2.35–2.53 (overlapping multiplets, 6H, (CH_2_)_3_-Me), 1.41–1.68, 3.60–3.73, 4.79–4.84 (overlapping multiplets, 9H, OTHP), 1.67 (s, 3H, Me-C), 4.46–4.61 (m, 2H, CH_2_O), 7.03–8.24 (m, 15H, 3Ph). ^13^C-NMR (150.9 MHz) δ = 14.2, 19.4, 23.2, 23.5 (*J =* 4.7 Hz), 25.4, 26.0 (*J =* 7.8 Hz), 31.1, 38.8 (*J =* 7.9 Hz), 62.4, 62.9 (*J =* 9.7 Hz), 95.7 (*J =* 10.0 Hz), 98.1 (*J =* 4.5 Hz), 111.2–138.5, 126.2 (*J =* 54.3 Hz), 189.4 (*J =* 74.3 Hz). ^31^P-NMR (242.9 MHz): δ 85.7. Anal. Calcd for C_32_H_38_ClO_3_PSe (616.03): C 62.39, H 6.22. Found: C 62.33, H 6.15.

*(1E)-3-Chloro-3-methyl-2-phenylselenenyl-1-[(tetrahydro-2H-pyran-2-yloxy)methyl]hept-1-en-1-yl diphenyl phosphine oxide* (**9c**). Yellow oil, yield: 24%. R_f_ 0.61; IR (neat, cm^−1^): 1120 (C-O-C), 1149 (P=O), 1440, 1493 (Ph), 1614 (C=C). ^1^H-NMR (600.1 MHz): δ 0.86 (t, *J =* 6.2 Hz, 3H, Me-CH_2_), 1.30–1.40, 1.44–1.69, 2.01–2.21, 3.36–3.74, 4.52–4.58 (overlapping multiplets, 15H, (CH_2_)_3_-Me), OTHP), 2.17 (s, 3H, Me-C), 4.07–4.21 (m, 2H, CH_2_O), 7.37–7.77 (m, 15H, 3Ph). ^13^C-NMR (150.9 MHz) δ = 14.1, 19.3, 22.7, 25.4, 26.8, 28.8 (*J =* 4.8 Hz), 31.0, 42.6 (*J =* 4.9 Hz), 62.4, 68.7 (*J =* 6.0 Hz), 80.5 (*J =* 7.9 Hz), 96.3 (*J =* 5.1 Hz), 128.4 (*J =* 105.3 Hz), 128.5–139.2, 154.2 (*J =* 85.2 Hz). ^31^P-NMR (242.9 MHz): δ 38.7. Anal. Calcd for C_32_H_38_ClO_3_PSe (616.03): C 62.39, H 6.22. Found: C 62.46, H 6.26.

*4-Bromo-2,2-diphenyl-3-[(tetrahydro-2H-pyran-2-yloxy)methyl]-1-oxa-2-phosphoniaspiro[4.5]dec-3-ene bromide* (**7d**). Orange oil, yield: 49%. R_f_ 0.35; IR (neat, cm^−1^): 1123 (C-O-C), 1435, 1490 (Ph), 1582 (C=C). ^1^H-NMR (600.1 MHz): δ 1.27–1.70, 1.99–2.05, 2.30–2.35, 3.60–3.77, 4.77–4.82 (overlapping multiplets, 15H, (CH_2_)_5_, OTHP), 4.38–4.50 (m, 2H, CH_2_O), 7.28–7.93 (m, 10H, 2Ph). 62.8, 88.6 (*J =* 9.9 Hz), 98.2 (*J =* 4.8 Hz), 110.8–133.8, 133.9 (*J =* 50.8 Hz), 157.7 (*J =* 49.0 Hz). ^31^P-NMR (242.9 MHz): δ 85.4. Anal. Calcd for C_26_H_31_Br_2_O_3_P (582.30): C 53.63, H 5.37. Found: C 53.70, H 5.32.

*(E)-2-bromo-2-(1-bromocyclohexyl)-1-[(tetrahydro-2H-pyran-2-yloxy)methyl]vinyl diphenyl phosphine oxide* (**9d**). Yellow oil, yield: 24%. R_f_ 0.62; IR (neat, cm^−1^): 1123 (C-O-C), 1173 (P=O), 1437, 1496 (Ph), 1620 (C=C). ^1^H-NMR (600.1 MHz): δ 1.26–1.37, 1.46–1.71, 2.00–2.19, 3.60–3.77, 4.54–4.59 (overlapping multiplets, 15H, (CH_2_)_5_, OTHP), 3.92–4.07 (m, 2H, CH_2_O), 7.51–8.10 (m, 15H, 3Ph). ^13^C-NMR (150.9 MHz) δ = 19.6, 22.1, 25.1, 25.5, 31.2, 39.6 (*J =* 5.0 Hz), 62.5, 68.2 (*J =* 5.8 Hz), 74.5 (*J =* 7.9 Hz), 96.1 (*J =* 5.0 Hz), 129.1–133.9, 132.2 (*J =* 154.7 Hz), 141.7 (*J =* 51.4 Hz). ^31^P-NMR (242.9 MHz): δ 37.2.Anal. Calcd for C_26_H_31_Br_2_O_3_P (582.30): C 53.63, H 5.37. Found: C 53.58, H 5.45.

*4-Bromo-2,2-diphenyl-3-[1-(tetrahydro-2H-pyran-2-yloxy)ethyl]-1-oxa-2-phosphoniaspiro[4.5]dec-3-ene bromide* (**7e**). Orange oil, yield: 49%. R_f_ 0.39; IR (neat, cm^−1^): 1118 (C-O-C), 1439, 1491 (Ph), 1591 (C=C). ^1^H-NMR (600.1 MHz): δ 1.29–1.73, 1.92–2.02, 2.27–2.33, 3.58–3.73, 4.91–4.95 (overlapping multiplets, 15H, (CH_2_)_5_, OTHP), 1.55 (d, 3H, *J =* 6.5 Hz, Me-CH), 4.30–4.39 (m, 1H, Me-CH), 7.31–7.89 (m, 10H, 2Ph). ^13^C-NMR (150.9 MHz) δ = 19.6, 22.1 (*J =* 4.8 Hz), 23.7, 25.3 (*J =* 7.7 Hz), 25.5, 30.9, 35.8 (*J =* 8.0 Hz), 62.4, 76.2 (*J =* 5.4 Hz), 89.4 (*J =* 9.8 Hz), 97.4 (*J =* 4.8 Hz), 111.2–134.0, 134.6 (*J =* 51.0 Hz), 156.8 (*J =* 48.3 Hz). ^31^P-NMR (242.9 MHz): δ 83.7. Anal. Calcd for C_27_H_33_Br_2_O_3_P (596.33): C 54.38, H 5.58. Found: C 54.45, H 5.64.

*(E)-2-bromo-2-(1-bromocyclohexyl)-1-[1-(tetrahydro-2H-pyran-2-yloxy)ethyl]vinyl diphenyl phosphine oxide* (**9e**). Dark orange oil, yield: 25%. R_f_ 0.64; IR (neat, cm^−1^): 1118 (C-O-C), 1165 (P=O), 1441, 1489 (Ph), 1621 (C=C). ^1^H-NMR (600.1 MHz): δ 1.26–1.37, 1.40–1.71, 1.98–2.16, 3.59–3.75, 4.64–4.69 (overlapping multiplets, 15H, (CH_2_)_5_, OTHP), 1.44 (dd, 3H, *J =* 6.5 Hz, *J =* 3.4 Hz, Me-CH), 4.78–4.86 (m, 1H, Me-CH), 7.50–8.04 (m, 10H, 2Ph). ^13^C-NMR (150.9 MHz) δ = 19.6, 21.8, 22.5 (*J =* 7.9 Hz), 25.3, 25.6, 31.3, 40.2 (*J =* 4.7 Hz), 62.4, 74.3 (*J =* 7.8 Hz), 81.4 (*J =* 5.0 Hz), 95.6 (*J =* 5.0 Hz), 129.4–134.5, 131.9 (*J =* 155.4 Hz), 142.3 (*J =* 49.7 Hz). ^31^P-NMR (242.9 MHz): δ 38.1. Anal. Calcd for C_27_H_33_Br_2_O_3_P (596.33): C 54.38, H 5.58. Found: C 54.32, H 5.54.

*2,2-Diphenyl-4-phenylselenenyl-3-[1-(tetrahydro-2H-pyran-2-yloxy)ethyl]-1-oxa-2-phosphoniaspiro-[4.5]dec-3-ene chloride* (**7f**). Dark orange oil, yield: 48%. R_f_ 0.36; IR (neat, cm^−1^): 1118 (C-O-C), 1436, 1488 (Ph), 1586 (C=C). ^1^H-NMR (600.1 MHz): δ 1.30–1.64, 1.67–1.78, 2.04–2.11, 3.58–3.74, 4.91–4.96 (overlapping multiplets, 15H, (CH_2_)_5_, OTHP), 1.48 (d, 3H, *J =* 6.4 Hz, Me-CH), 4.18–4.26 (m, 1H, Me-CH), 7.28–7.91 (m, 15H, 3Ph). ^13^C-NMR (150.9 MHz) δ = 19.5, 21.2 (*J =* 5.1 Hz), 23.5 (*J =* 7.9 Hz), 23.6, 25.6, 31.4, 34.8 (*J =* 7.9 Hz), 62.3, 76.8 (*J =* 5.3 Hz), 92.4 (*J =* 9.8 Hz), 97.9 (*J =* 4.9 Hz), 111.1–138.6, 131.4 (*J =* 50.7 Hz), 176.7 (*J =* 88.5 Hz). ^31^P-NMR (242.9 MHz): δ 86.5. Anal. Calcd for C_33_H_38_ClO_3_PSe (628.04): C 63.11, H 6.10. Found: C 63.18, H 6.16.

*(E)-2-(1-chlorocyclohexyl)-2-phenylselenenyl-1-[1-(tetrahydro-2H-pyran-2-yloxy)ethyl]vinyl diphenyl phosphine oxide* (**9f**). Light orange oil, yield: 24%. R_f_ 0.62; IR (neat, cm^−1^): 1118 (C-O-C), 1167 (P=O), 1438, 1490 (Ph), 1621 (C=C). ^1^H-NMR (600.1 MHz): δ 1.28–1.77, 1.99–2.18, 3.59–3.76, 4.63–4.69 (overlapping multiplets, 15H, (CH_2_)_5_, OTHP), 1.36 (dd, 3H, *J =* 6.5 Hz, *J =* 3.6 Hz, Me-CH), 4.38–4.45 (m, 1H, Me-CH), 7.37–7.74 (m, 15H, 3Ph). ^13^C-NMR (150.9 MHz) δ = 19.6, 20.7, 21.0 (*J =* 7.8 Hz), 25.6, 25.7, 31.2, 38.4 (*J =* 4.6 Hz), 62.3, 71.4 (*J =* 7.9 Hz), 81.3 (*J =* 4.8 Hz), 95.7 (*J =* 4.8 Hz), 128.4–139.4, 131.3 (*J =* 105.4 Hz), 152.3 (*J =* 71.5 Hz). ^31^P-NMR (242.9 MHz): δ 36.0. Anal. Calcd for C_33_H_38_ClO_3_PSe (628.04): C 63.11, H 6.10. Found: C 63.07, H 6.05.

*5-Butyl-4-chloro-5-methyl-3-[1-methyl-1-(tetrahydro-2H-pyran-2-yloxy)ethyl]-2,2-diphenyl-2,5-dihydro-1,2-oxaphosphol-2-ium chloride* (**7g**). Yellow oil, yield: 54%. R_f_ 0.38; IR (neat, cm^−1^): 1119 (C-O-C), 1435, 1484 (Ph), 1582 (C=C). ^1^H-NMR (600.1 MHz): δ 0.91 (t, *J =* 6.4 Hz, 3H, Me-CH_2_), 1.15–1.21, 1.27–1.34, 2.28–2.41 (overlapping multiplets, 6H, (CH_2_)_3_-Me), 1.42–1.65, 3.69–3.84, 4.97–5.02 (overlapping multiplets, 9H, OTHP), 1.68 (s, 3H, Me-C), 1.70 (s, 6H, Me_2_C), 7.63–8.26 (m, 10H, 2Ph). ^13^C-NMR (150.9 MHz) δ = 14.1, 20.5, 23.2, 23.8 (*J =* 4.5 Hz), 25.3, 26.1 (*J =* 7.8 Hz), 29.7 (*J =* 8.1 Hz), 32.3, 39.1 (*J =* 7.8 Hz), 63.7, 79.5 (*J =* 9.8 Hz), 92.7 (*J =* 9.7 Hz), 95.7 (*J =* 4.7 Hz), 106.5–134.6, 133.4 (*J =* 50.2 Hz), 164.8 (*J =* 40.5 Hz). ^31^P-NMR (242.9 MHz): δ 82.0. Anal. Calcd for C_28_H_37_Cl_2_O_3_P (523.47): C 64.24, H 7.12. Found: C 64.19, H 7.05.

*(1E)-2,3-dichloro-3-methyl-1-[1-methyl-1-(tetrahydro-2H-pyran-2-yloxy)ethyl]hept-1-en-1-yl diphenyl phosphine oxide* (**9g**). Orange oil, yield: 25%. R_f_ 0.63; IR (neat, cm^−1^): 1119 (C-O-C), 1159 (P=O), 1439, 1485 (Ph), 1617 (C=C). ^1^H-NMR (600.1 MHz): δ 0.87 (t, *J =* 6.3 Hz, 3H, Me-CH_2_), 1.35–1.48, 1.54–1.69, 2.27–2.54 (overlapping multiplets, 6H, (CH_2_)_3_-Me), 1.47–1.68, 3.66–3.80, 4.70–4.76 (overlapping multiplets, 9H, OTHP), 1.58 (s, 3H, Me_2_C), 1.83 (s, 3H, Me-C), 7.53–7.91 (m, 10H, 2Ph). ^13^C-NMR (150.9 MHz) δ = 14.0, 20.6, 22.9, 25.2, 26.4, 29.5 (*J =* 4.7 Hz), 30.8 (*J =* 7.9 Hz), 32.2, 42.9 (*J =* 4.7 Hz), 63.6, 76.4 (*J =* 7.8 Hz), 80.3 (*J =* 9.9 Hz), 93.4 (*J =* 4.7 Hz), 129.3–134.2, 133.6 (*J =* 101.4 Hz), 152.9 (*J =* 41.2 Hz). ^31^P-NMR (242.9 MHz): δ 37.7. Anal. Calcd for C_28_H_37_Cl_2_O_3_P (523.47): C 64.24, H 7.12. Found: C 64.28, H 7.20.

### 3.6. General Procedure for the Reactions of the 2-Diphenylphosphinoyl-2,3-dien-1-ols **4a**–**c** and 3-Diphenylphosphinoyl-3,4-dien-2-ols **4d**,**e** with Electrophilic Reagents

To a solution of the 2-diphenylphosphinoyl-2,3-dien-1-ols **4a**–**c** or the 3-diphenylphosphinoyl-3,4-dien-2-ols **4d**,**e** (3.0 mmol) in dry dichloromethane(10 mL) at −20 °C was added dropwise with stirring a solution of electrophilic reagent (sulfuryl chloride, bromine, benzenesulfenyl chloride, benzeneselenenyl chloride) (3.6 mmol) in the same solvent (10 mL). The reaction mixture was stirred at the same temperature for several hours (see Table 2) and an hour at room temperature. The solvent was removed using a rotatory evaporator and the residue was purified by column chromatography (silica gel, ethyl acetate and hexane 2:1). The pure products **8** and **10** had the following properties:

*4-Bromo-5-ethyl-3-(hydroxymethyl)-5-methyl-2,2-diphenyl-2,5-dihydro-1,2-oxaphosphol-2-ium bromide* (**8a**). Pale orange oil, yield: 52%. R_f_ 0.38; IR (neat, cm^−1^): 1435, 1485 (Ph), 1581 (C=C), 3375 (OH). ^1^H-NMR (600.1 MHz): δ 1.08 (t, *J =* 7.3 Hz, 3H, Me-CH_2_), 1.80 (s, 3H, Me-C), 2.31–2.40 (m, 2H, Me-CH_2_), 4.57 (s, 1H, OH), 4.95–5.02 (m, 2H, CH_2_O), 7.80–8.45 (m, 10H, 2Ph). ^13^C-NMR (150.9 MHz) δ = 8.1 (*J =* 4.6 Hz), 27.2 (*J =* 7.8 Hz), 31.0 (*J =* 7.7 Hz), 60.2 (*J =* 5.8 Hz), 92.7 (*J =* 9.7 Hz), 111.5–135.1, 133.7 (*J =* 49.7 Hz), 158.1 (*J =* 50.3 Hz). ^31^P-NMR (242.9 MHz): δ 88.1. Anal. Calcd for C_19_H_21_Br_2_O_2_P (472.15): C 48.33, H 4.48. Found: C 48.26, H 4.55.

*(2E)-3,4-Dibromo-2-diphenylphosphinoyl-4-methylhex-2-en-1-ol* (**10a**). Yellow oil, yield: 23%. R_f_ 0.64; IR (neat, cm^−1^): 1171 (P=O), 1433, 1482 (Ph), 1628 (C=C), 3374 (OH). ^1^H-NMR (600.1 MHz): δ 1.14 (t, *J =* 7.3 Hz, 3H, Me-CH_2_), 1.98–2.18 (m, 2H, Me-CH_2_), 2.18 (s, 3H, Me-C), 2.98 (s, 1H, OH), 4.53–4.57 (m, 2H, CH_2_O), 7.53–8.08 (m, 10H, 2Ph). ^13^C-NMR (150.9 MHz) δ = 9.2, 35.2 (*J =* 5.0 Hz), 36.4 (*J =* 5.0 Hz), 62.0 (*J =* 7.9 Hz), 63.6 (*J =* 5.9 Hz), 129.4–133.7, 131.5 (*J =* 49.9 Hz), 141.7 (*J =* 50.8 Hz). ^31^P-NMR (242.9 MHz): δ 39.6. Anal. Calcd for C_19_H_21_Br_2_O_2_P (472.15): C 48.33, H 4.48. Found: C 48.40, H 4.52.

*5-Butyl-3-(hydroxymethyl)-5-methyl-2,2-diphenyl-4-phenylselenenyl-2,5-dihydro-1,2-oxaphosphol-2-ium chloride* (**8b**). Yellow oil, yield: 46%. R_f_ 0.39; IR (neat, cm^−1^): 1438, 1487 (Ph), 1585 (C=C), 3380 (OH). ^1^H-NMR (600.1 MHz): δ 0.92 (t, *J =* 7.2 Hz, 3H, Me-CH_2_), 1.02–1.11, 1.27–1.37, 2.35–2.54 (m, 6H, (CH_2_)_3_-Me), 1.67 (s, 3H, Me-C), 5.08–5.13 (m, 2H, CH_2_O), 5.24 (s, 1H, OH), 6.99–8.28 (overlapping multiplets, 15H, 3Ph). ^13^C-NMR (150.9 MHz) δ = 13.8, 22.1 (*J =* 5.1 Hz), 23.4, 26.7 (*J =* 7.9 Hz), 38.5 (*J =* 7.9 Hz), 60.5 (*J =* 5.8 Hz), 95.8 (*J =* 9.9 Hz), 111.4–138.6, 126.2 (*J =* 51.3 Hz), 189.0 (*J =* 69.4 Hz). ^31^P-NMR (242.9 MHz): δ 86.9. Anal. Calcd for C_27_H_30_ClO_2_PSe (531.91): C 60.97, H 5.68. Found: C 60.92, H 5.73.

*(2E)-4-Chloro-2-diphenylphosphinoyl-4-methyl-3-phenylselenenyl-oct-2-en-1-ol* (**10b**). Yellow oil, yield: 26%. R_f_ 0.65; IR (neat, cm^−1^): 1175 (P=O), 1437, 1490 (Ph), 1620 (C=C), 3389 (OH). ^1^H-NMR (600.1 MHz): δ 0.88 (t, *J =* 7.1 Hz, 3H, Me-CH_2_), 1.30–1.46, 2.36–2.63 (m, 6H, (CH_2_)_3_-Me), 1.79 (s, 3H, Me-C), 3.74 (s, 1H, OH), 4.67–4.72 (m, 2H, CH_2_O), 7.35–7.98 (overlapping multiplets, 15H, 3Ph). ^13^C-NMR (150.9 MHz) δ = 14.1, 22.9, 25.7, 28.9 (*J =* 4.8 Hz), 42.4 (*J =* 4.7 Hz), 64.7 (*J =* 6.0 Hz), 80.0 (*J =* 7.9 Hz), 127.7 (*J =* 101.4 Hz), 129.0–139.1, 153.6 (*J =* 57.4 Hz). ^31^P-NMR (242.9 MHz): δ 39.9. Anal. Calcd for C_27_H_30_ClO_2_PSe (531.91): C 60.97, H 5.68. Found: C 61.02, H 5.75.

*4-Chloro-3-(hydroxymethyl)-2,2-diphenyl-1-oxa-2-phosphonia-spiro[4.5]dec-3-ene chloride* (**8c**). Yellow oil, yield: 54%. R_f_ 0.37; IR (neat, cm^−1^): 1441, 1489 (Ph), 1582 (C=C), 3389 (OH). ^1^H-NMR (600.1 MHz): δ 1.30–1.49, 1.61–1.88, 2.18–2.24 (overlapping multiplets, 10H, (CH_2_)_5_), 4.99–5.04 (m, 2H, CH_2_O), 5.30 (s, 1H, OH), 7.75–8.31 (m, 10H, 2Ph). ^13^C-NMR (150.9 MHz) δ = 22.5 (*J =* 4.6 Hz), 24.0, 35.6 (*J =* 7.8 Hz), 60.3 (*J =* 5.8 Hz), 90.3 (*J =* 9.8 Hz), 106.3–133.8, 127.1 (*J =* 49.7 Hz), 171.4 (*J =* 42.6 Hz). ^31^P-NMR (242.9 MHz): δ 86.5. Anal. Calcd for C_21_H_23_Cl_2_O_2_P (409.29): C 61.63, H 5.66. Found: C 61.70, H 5.71.

*(2E)-3-Chloro-3-(1-chlorocyclohexyl)-2-diphenylphosphinoyl-prop-2-en-1-ol* (**10c**). Pale orange oil, yield: 26%. R_f_ 0.62; IR (neat, cm^−1^): 1178 (P=O), 1440, 1493 (Ph), 1619 (C=C), 3384 (OH). ^1^H-NMR (600.1 MHz): δ 1.28–1.41, 1.55–1.70, 1.76–1.92 (overlapping multiplets, 10H, (CH_2_)_5_), 3.77 (s, 1H, OH), 4.58–4.63 (m, 2H, CH_2_O), 7.43–7.99 (m, 10H, 2Ph). ^13^C-NMR (150.9 MHz) δ = 21.8, 25.7, 38.5 (*J =* 5.1 Hz), 63.7 (*J =* 5.9 Hz), 68.3 (*J =* 9.9 Hz), 129.1 (*J =* 100.9 Hz), 129.5–134.4, 148.7 (*J =* 39.7 Hz). ^31^P-NMR (242.9 MHz): δ 34.6. Anal. Calcd for C_21_H_23_Cl_2_O_2_P (409.29): C 61.63, H 5.66. Found: C 61.69, H 5.60.

*3-(1-Hydroxyethyl)-2,2-diphenyl-4-phenylsulfenyl-1-oxa-2-phosphonia-spiro[4.5]dec-3-ene chloride* (**8d**). Yellow oil, yield: 45%. R_f_ 0.38; IR (neat, cm^−1^): 1435, 1494 (Ph), 1580 (C=C), 3393 (OH). ^1^H-NMR (600.1 MHz): δ 1.31–1.47, 1.71–1.97, 2.07–2.13 (overlapping multiplets, 10H, (CH_2_)_5_), 1.78 (dd, *J =* 16.6 Hz,*J =* 6.6 Hz, 3H, Me-CH), 4.32 (s, 1H, OH), 4.96–5.07 (m, 1H, Me-CH), 6.91–8.60 (overlapping multiplets, 15H, 3Ph). ^13^C-NMR (150.9 MHz) δ = 22.8 (*J =* 5.1 Hz), 23.6, 26.2 (*J =* 7.9 Hz), 35.8 (*J =* 7.8 Hz), 70.6 (*J =* 5.1 Hz), 89.9 (*J =* 9.9 Hz), 125.6–139.1, 133.4 (*J =* 51.0 Hz), 164.7 (*J =* 15.1 Hz). ^31^P-NMR (242.9 MHz): δ 86.0. Anal. Calcd for C_28_H_30_ClO_2_PS (497.03): C 67.66, H 6.08. Found: C 67.71, H 6.12.

*(3E)-4-(1-Chlorocyclohexyl)-3-diphenylphosphinoyl-4-phenylsulfenyl-but-3-en-2-ol* (**10d**). Orange oil, yield: 25%. R_f_ 0.62; IR (neat, cm^−1^): 1169 (P=O), 1441, 1488 (Ph), 1618 (C=C), 3391 (OH). ^1^H-NMR (600.1 MHz): δ 1.34–1.46, 1.49–1.54, 1.59–1.78 (overlapping multiplets, 10H, (CH_2_)_5_), 1.34 (dd, *J =* 15.3 Hz,*J =* 6.5 Hz, 3H, Me-CH), 3.88 (s, 1H, OH), 4.63–4.74 (m, 1H, Me-CH), 7.36–7.71 (overlapping multiplets, 15H, 3Ph). ^13^C-NMR (150.9 MHz) δ = 22.7, 23.4 (*J =* 7.9 Hz), 25.6, 38.9 (*J =* 4.6 Hz), 68.1 (*J =* 7.9 Hz), 76.3 (*J =* 5.0 Hz), 126.5–137.4, 133.2 (*J =* 101.0 Hz), 162.4 (*J =* 15.0 Hz). ^31^P-NMR (242.9 MHz): δ 34.0.Anal. Calcd for C_28_H_30_ClO_2_PS (497.03): C 67.66, H 6.08. Found: C 67.59, H 6.13.

*5-Butyl-3-(1-hydroxy-1-methylethyl)-5-methyl-2,2-diphenyl-4-phenylselenenyl-2,5-dihydro-1,2-oxa-phosphol-2-ium chloride* (**8e**). Yellow oil, yield: 46%. R_f_ 0.40; IR (neat, cm^−1^): 1438, 1487 (Ph), 1585 (C=C), 3393 (OH). ^1^H-NMR (600.1 MHz): δ 0.88 (t, *J =* 7.1 Hz, 3H, Me-CH_2_), 1.03–1.11, 1.27–1.34, 2.36–2.50 (m, 6H, (CH_2_)_3_-Me), 1.58 (s, 3H, Me_2_C), 1.64 (s, 3H, Me-C), 5.24 (s, 1H, OH), 6.94–8.14 (overlapping multiplets, 15H, 3Ph). ^13^C-NMR (150.9 MHz) δ = 14.1, 22.1 (*J =* 5.1 Hz), 23.4, 26.4 (*J =* 7.8 Hz), 32.7 (*J =* 8.0 Hz), 38.4 (*J =* 7.8 Hz), 81.4 (*J =* 9.7 Hz), 97.4 (*J =* 9.8 Hz), 112.7–138.4, 135.3 (*J =* 53.7 Hz), 178.0 (*J =* 69.4 Hz). ^31^P-NMR (242.9 MHz): δ 89.4. Anal. Calcd for C_29_H_34_ClO_2_PSe (559.97): C 62.20, H 6.12. Found: C 62.26, H 6.05.

*(3E)-5-Chloro-3-diphenylphosphinoyl-2,5-dimethyl-4-phenylselenenyl-non-3-en-2-ol* (**10e**). Yellow oil, yield: 24%. R_f_ 0.65; IR (neat, cm^−1^): 1170 (P=O), 1438, 1486 (Ph), 1625 (C=C), 3396 (OH). ^1^H-NMR (600.1 MHz): δ 0.89 (t, *J =* 7.3 Hz, 3H, Me-CH_2_), 1.30–1.44, 2.38–2.61 (m, 6H, (CH_2_)_3_-Me), 1.46, 1.48 (ss, 3H, Me_2_C), 1.77 (s, 3H, Me-C), 4.13 (s, 1H, OH), 7.38–7.69 (overlapping multiplets, 15H, 3Ph). ^13^C-NMR (150.9 MHz) δ = 14.1, 22.9, 25.48, 29.3 (*J =* 5.1 Hz), 29.9 (*J =* 7.8 Hz), 43.3 (*J =* 4.8 Hz), 82.3 (*J =* 7.8 Hz), 82.8 (*J =* 9.9 Hz), 128.4–139.1, 135.8 (*J =* 102.7 Hz), 153.7 (*J =* 15.1 Hz). ^31^P-NMR (242.9 MHz): δ 36.9. Anal. Calcd for C_29_H_34_ClO_2_PSe (559.97): C 62.20, H 6.12. Found: C 62.25, H 6.08.

## 4. Conclusions

In conclusion, a simple and convenient protocol for the reaction of the phosphorylated α-hydroxyallenes with protected or unprotected hydroxy groups with different electrophilic reagents was developed. It involves a 5-*endo-trig* cyclization and 2,3-addition reactions depending on the substituents on the phosphoryl group. Treatment of 1-hydroxyalkyl-1,2-dienephosphonates with electrophiles gives 2-methoxy-2-oxo-2,5-dihydro-1,2-oxaphospholes as a result of participation of the phosphonate group in the cyclization. On the other hand, (1*E*)-alk-1-en-1-yl phosphine oxides were prepared as mixtures with 2,5-dihydro-1,2-oxaphosphol-2-ium halides in a ratio of about 1:2 by chemo-, regio, and stereoselective electrophilic addition to the C^2^-C^3^-double bond in the allene moiety and subsequent concurrent attack of the external (halide anion) and internal (phosphine oxide group) nucleophiles.

Thanks to the ready availability of the starting materials, the convenient operation and the usefulness of the resulting 1,2-oxaphosphole products this reaction show great potential and will be useful in organic synthesis. Further studies on the synthetic applications of this reaction and the physiological activity of selected cyclic and acyclic products, and extension of these studies to the synthesis and electrophilic cyclization and cycloisomerization reactions of other bifunctionalized allenes is currently in progress in our laboratory.
